# Supplementary material for: The genome sequence of a plutellid moth, Plutella hyperboreella Strand, 1902 (Lepidoptera: Plutellidae)
Source: Wellcome Open Res. 2026 Sep 11;11:628. [Version 1] doi: 10.12688/wellcomeopenres.27772.1 (PMC13613003; doi:10.12688/wellcomeopenres.27772.1)
Supplement: Peer review history

# Reviewer report: Reviewer response for version 1

**DOI:** 10.21956/wellcomeopenres.30563.r169446

**Published:** 2026-09-24

**Authors:** Will Nash

**Affiliations:** 1 University of East Anglia School of Biological Sciences (Ringgold ID: 98950), Norwich, England, UK

The manuscript presents two haplotype assemblies of the moth *Plutella hyperboreella*, spanning 503.34 Mb & 426.42 Mb respectively. Hap 1 is assigned to 31 chromosome pseudomolecules. 12,347 protein coding genes are also annotated based on computational inference.

 The background is well written and presents a strong rationale for studying the target species, based on cold adaptation, as well as links to pest species.

 All methods are appropriate and current for generating a high-quality genome from a single small insect specimen. Wet lab methods are well documented through protocols.io, with specific modifications for the species also described. I am happy to see yields and fragment sizes included. Detailed library prep (PacBio and Hi-C) methodologies are included – this is great and extends reproducibility for small insect studies. Bioinformatic methods are very clear and well documented.

 The sequencing report shows 3.96 million HiFi reads were used to assemble the genome. The k-mer spectrum shows a clear diploid profile, with a well-fitted GenomeScope model (96.47%), an estimated heterozygosity of 1.02%, and ample HiFi sequencing depth (~76×) for haplotype-resolved assembly. Assembly stats (98.75% chromosome-assigned, QV 61.8, BUSCO 99.3%) suggest a very strong assembly following scaffolding. The genome is computationally annotated, with 12,347 protein-coding genes, with an average transcript length of ~13 kb. It would be useful for the authors to state more explicitly what evidence sources were incorporated into the Ensembl annotation, particularly whether transcriptomic evidence was available. RNA-seq-supported annotation would provide additional confidence in gene models, although I recognise that obtaining suitable RNA material can be challenging for small insect specimens and may be beyond the scope of this Genome Note.

 Overall, this is a well-produced and highly reproducible genome resource. I have only a small number of comments that I think would improve clarity:

1. **Difference in haplotype assembly span.** Haplotype 1 is 503.34 Mb whereas haplotype 2 is 426.42 Mb. The W and Z chromosomes in haplotype 1 are 39.58 Mb and 37.75 Mb, respectively, together accounting for ~77.3 Mb, very close to the ~76.9 Mb difference between the two haplotype assemblies. This appears biologically consistent with the female WZ specimen and the representation of both sex chromosomes within haplotype 1. I suggest that the authors add a short explanation of the difference in assembly span, as it is otherwise quite striking when first encountering Table 2.
2. **Individual haplotype k-mer completeness.** K-mer completeness is reported as 82.80% for haplotype 1 and 74.70% for haplotype 2, while the combined assembly reaches 99.41%. I think a sentence explaining the expected interpretation of the individual versus combined haplotype values would help readers, particularly given the phased nature of the assembly and the sex chromosomes.
3. **Sex chromosome identification.** The manuscript states that the W and Z chromosomes were identified using copy number in the diploid assembly. It would be useful to provide a little more detail on the coverage/copy-number evidence used for these assignments.

Are the datasets clearly presented in a usable and accessible format, and the assembly and annotation available in an appropriate subject-specific repository?

Yes

Are sufficient details of the sequencing and extraction, software used, and materials provided to allow replication by others?

Yes

Are the rationale for sequencing the genome and the species significance clearly described?

Yes

Are the protocols appropriate and is the work technically sound?

Yes

Reviewer Expertise:

Entomology, Population Genomics, Comparative Genomics, Genome Assembly,

I confirm that I have read this submission and believe that I have an appropriate level of expertise to confirm that it is of an acceptable scientific standard.

# Reviewer report: Reviewer response for version 1

**Published:** 2026-09-11

**Authors:** Automated Benchmarking

**Affiliations:** 1 Wellcome Sanger Institute, Hinxton, UK

As this article is a Genome Note, Automated Benchmarking has been used to determine whether the reported assembly passes three defined checks established by the Earth BioGenome Project: the raw and assembled data are publicly available, the Earth BioGenome Project three-part metric is met, and the BUSCO score passes the stated threshold. For Plutella hyperboreella (ilPluHype1), all three checks passed. Data available: pass. ENA study PRJEB92268, raw-read accessions ERR15282569 and ERR15286553, and assembly accessions GCA_966184305.1 and GCA_966185215.1 are public. Earth BioGenome Project three-part metric: pass. Haplotype 1 is reported as 7.C.Q61, meeting the 6.C.Q40 reference standard. BUSCO: pass. Haplotype 1, which is proposed as the reference assembly for this species, has 99.3% complete BUSCOs (97.5% single-copy and 1.8% duplicated). It meets the target of greater than 90% complete and single-copy BUSCOs. This decision uses values reported in the Genome Note and public accession records.

Reviewer Expertise:

NA

I confirm that I have read this submission and believe that I have an appropriate level of expertise to confirm that it is of an acceptable scientific standard.
